# Multiple Associated Anomalies In Patients Of Duodenal Atresia: A Case Series

**Published:** 2012-04-01

**Authors:** Bilal Mirza, Afzal Sheikh

**Affiliations:** Department of Paediatric Surgery, The Children's Hospital and the Institute of Child Health Lahore, Pakistan

**Keywords:** Duodenal atresia, multiple anomalies, Down syndrome, malrotation, annular pancreas

## Abstract

Duodenal atresia has been reported in association with various malformations and syndromes common being Down syndrome, malrotation, and annular pancreas. Its association with multiple anomalies is rare and scarcely reported in literature. Herein 3 cases of duodenal atresia associated with multiple congenital anomalies are being reported.

## INTRODUCTION

Duodenal atresia is the frequent cause of neonatal intestinal obstruction that has gained substantial importance since its first description by Calder in 1733 [1]. The prognosis was initially poor, but it improved peculiarly with the advent of modern anesthesia, better understanding of pathophysiology, and intensive care units. The survival is quite promising (>90%) in the entity with exception of very few cases where mortality is attributed to associated anomalies especially complex cardiac anomalies [1-4].

In 30% of patients, it is associated with Down syndrome. Malrotation, annular pancreas, and Meckel’s diverticulum etc. are other associated anomalies. Its association with multiple anomalies is however very rare and scarcely reported in literature [1-4]. Extreme rarity of multiple anomalies associated with duodenal atresia prompted us to report these neonates.

## DESCRIPTION

The mean age of presentation was 2.3 days (SD ±0.57). There were 2 males and one female. Mean weight was 2.3kg (SD ±0.3). All three presented with bilious vomiting and intolerance to the feeds. All the patients had typical features of Down syndrome. Radiographs showed double-bubble sign in all patients. Clotting profile was deranged in 2 patients. Rest of laboratory investigations were within reference range in all patients. All patients were resuscitated in the neonatal emergency department with intravenous fluids, vitamin K, and prophylactic antibiotics. Deranged clotting profile was corrected in affected cases by infusing fresh frozen plasma. At surgery, 2 patients had type-I duodenal atresia (Fig. 1), whereas in 1 patient type-III duodenal atresia was found. Malrotation was present in all patients. Other anomalies in these patients were annular pancreas in 1 patient and Meckel’s diverticulum in another patient. Diamond shaped duodenoduodenostomy and Ladd’s procedure was performed in all patients (Table 1). Postoperative recovery was uneventful in 2 patients. One patient had a stormy postoperative course. He had prolonged duodenal ileus and was put on total parental nutrition; however, succumbed to sepsis on 13th postoperative day. Table 1 shows the summary of these patients.

**Figure:**
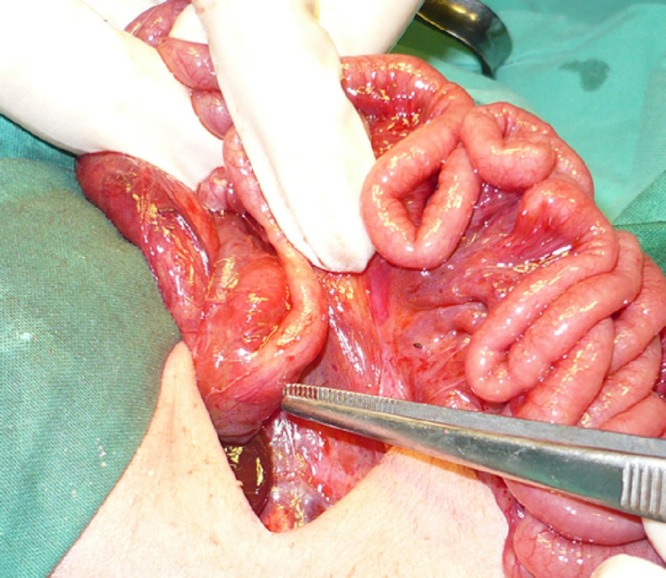
Figure 1: Duodenal atresia

**Figure:**
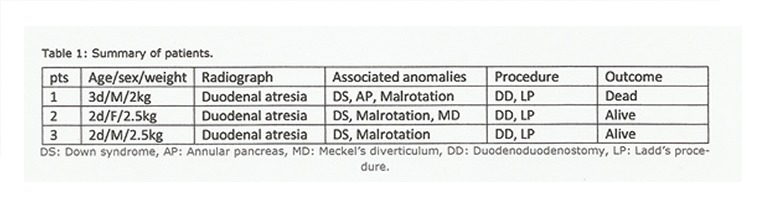
Table 1: Summary of patients

## DISCUSSION

In more than half of the patients with duodenal atresia, associated anomalies and syndromes are present. Down syndrome is present in 30% of cases, malrotation in 20%, and congenital heart diseases in 20% of cases; nevertheless, other congenital anomalies of alimentary tract are also present in these patients. In 2/3rd patients, the associated anomalies occur in isolation, whereas multiple anomalies occur in 1/3rd of patients. Although presence of Down syndrome in patients of duodenal atresia does not affect survival, the presence of multiple anomalies may alter the final outcome. Escobar et al found 6% of late mortality attributed to associated anomalies, central nervous system bleeding, pneumonia, and anastomotic disruption etc [2,3].

We have had reported a case of duodenal atresia associated with Down syndrome, malrotation, and annular pancreas [4]. The similar case was then reported by Gonçalves et al [5]. In the previously reported case, we could not evaluate the patient for trisomy-21. Gonçalves et al documented trisomy-21 in their patient [5]. In our series, one patient was evaluated by karyotyping that confirmed trisomy-21, while the other patient was lost to follow-up. The repetition of this specific set of congenital anomalies in association with duodenal atresia may point a new syndrome, or it may be a result of the same chromosomal aberration that constitutes Down syndrome. Further chromosomal studies are suggested in this regard.

## Footnotes

**Source of Support:** Nil

**Conflict of Interest:** None declared

